# Perspectives on Social Suffering in Interviews and Drawings of Palestinian Adults Crossing the Qalandia Checkpoint: A Qualitative Phenomenological Study

**DOI:** 10.3389/fpsyg.2018.01591

**Published:** 2018-08-28

**Authors:** Nihal M. Nagamey, Limor Goldner, Rachel Lev-Wiesel

**Affiliations:** The Emili Sagol Creative Arts Therapies Research Center, The School of Creative Art Therapies, University of Haifa, Haifa, Israel

**Keywords:** humiliation, social suffering, war and conflicts, Palestinians, projective drawings, checkpoints

## Abstract

The current study examined the psychological experience of Palestinians who daily cross an Israel Defense Forces (IDF) checkpoint to reach their schools or places of employment. The study employed an interpretative phenomenological analysis of semi-structured interviews and drawings to capture a depth insight regarding the psychological meaning of crossing the Qalandia checkpoint on a daily basis among 20 adult participants (10 males, 10 females). Three themes emerged. The first theme described deep feelings of distress and desperation and included the categories of humiliation and dehumanization, non-existence, rage, and pessimism and helplessness. The second theme concentrated on the participants’ coping strategies of avoidance and dissociation, which usually characterize maladaptive trauma coping style, as well as exhibited aggressiveness toward their fellow community members, while the third theme described the social fragmentation of the Palestinians’ solidarity. Furthermore, three pictorial phenomena emerged from the participants’ drawings: squared restricted drawings, the use of multiple black tiny objects, and the use of split drawings. These phenomena supported and validated participants’ verbal expressions. We suggest understanding these findings in light of the term “social suffering.”

## Introduction

Hundreds of millions of people around the world are affected by political conflicts that create widespread suffering, experiencing a wide range of psychological and mental symptoms (Veronese and Barola, 2018). One of these conflicts is the Israeli-Palestinian conflict, which has existed for most of the past century and endures with no foreseeable resolution (McNeely et al., 2014). The term *Palestinians* refers to “the people who lived in British Mandate Palestine before 1948, when the state of Israel was established, and their descendants.” (3, p. 837). Estimates indicate that three-quarters of the Palestinian population were dispossessed between 1947 and 1949 to neighboring Arab states, becoming refugees (Pappe, 2006; Rogan and Shlaim, 2007). This traumatic ejection, which is called by the Palestinians “the Nakba" (or catastrophe), is deep-rooted in the collective memory of the Palestinians and is still felt by third-generation refugees, especially those living in refugee camps (Baker and Shalhoub-Kevorkian, 1999).

Living under conditions of chronic conflict and a generations-long history of political turmoil and suffering in the occupied Palestinian territories (Gerner, 1991; Giacaman et al., 2009; B’Tselem, 2018) has led to substantial impairment in individuals’ mental and physical health among Palestinian adults (e.g., (Giacaman et al., 2004, 2007a,c; Barber and Schluterman, 2009; Rabaia et al., 2010). These have been manifested in a wide range of symptoms of anxiety and depression, including sleeplessness, uncontrollable fear, fatigue, and hopelessness (Giacaman et al., 2004), as well as health complaints including emotional and somatic scales (Abdeen et al., 2008; Giacaman et al., 2011) and PTSD symptoms (Abdeen et al., 2008). These symptoms were magnified by exposure to warlike events, such as the sounds of bombs, tear gas, shooting, and physical harm and humiliation (Giacaman et al., 2007a,c; Abdeen et al., 2008), creating feelings of being mentally exhausted, broken, and destroyed and hampering a sense of security and safety for one’s self, family, and home, (McNeely et al., 2014).

Rejecting the use of psychopathology categorizations, recently, scholars have moved from investigating psychopathology into studying Palestinians’ subjective well-being and quality of life under the contextual conditions of social, psychological, political, and economic threats and human insecurity (Giacaman et al., 2007b, 2009; Mataria et al., 2009). According to this approach, clinical language fails to capture the range and nuances of experience related to Palestinians’ well-being. Thus, a contextual approach that takes into account aspects of humiliation and social suffering within an ease-disease continuum ranging from mental well-being to mental disease should be adopted (Giacaman et al., 2007b, 2009, 2011). In general, quantitative studies have demonstrated severe impairments in Palestinians’ subjective well-being and quality of life (Mataria et al., 2009; Harsha et al., 2016). Perceptions of social instability, humiliation, and psychological suffering have been associated with lower levels of well-being (Giacaman et al., 2007a; Veronese et al., 2017). Qualitative research that concentrated on Palestinians’ quality of life revealed a holistic and fluctuating characterization of mental and physical well-being, including reports of a lack of happiness, diminishing energy and competency, a sense of fragmentation, physical complaints, and illness (Giacaman et al., 2011). The Israeli military occupation, including the severe movement restrictions, and poor living conditions were top determinants of Palestinians’ well-being, because of the daily problems they caused. Political freedom and participating and being involved in democratic processes were considered as essential contributors to Palestinians’ quality of life (Giacaman et al., 2007b). These studies underscore the importance to further explore the subjective experience of Palestinian well-being. Following this proposal, in the current study, we explored Palestinian’s well-being in the context of the checkpoints.

### Checkpoints: Definition

The UN defines checkpoints as any staffed physical impediment or barrier to travel within a territory. In the Israeli-Palestinian context, checkpoints refer to a subset of a broader infrastructure of closures within the West Bank, whose purpose is to restrict movement of people and resources within the Palestinian territory and between Palestinian communities (Giacaman et al., 2011). They are different than “crossings,” which delimit the border between the Palestinian territories and Israel. Checkpoints vary in material, form, placement, and function and impose different requirements for passage (Giacaman et al., 2011; Harsha et al., 2016). However, movement is only possible using a pass system, which identifies every Palestinian by mechanisms such as a color code or a biometric identification card that can be applied for (Veronese et al., 2017).

### Historical Background Regarding Checkpoints in Israel

Restrictions on Palestinian mobility in the West Bank have existed since 1967 with Israel occupying the rest of historical Palestine territories, including the West Bank, East Jerusalem, and the Gaza Strip (Giacaman et al., 2009). These restrictions were established by the IDF and were a vital aspect of the first Intifada (the first popular resistance against the Israeli military occupation) during 1987–1993 (Longo et al., 2014) which led to a series of increasingly controlling acts such including school and university closures, destruction of houses, exiles, land seizures, and arrests (Gerner, 1991; B’Tselem, 2018).

An official policy of restriction of Palestinian movement in the West Bank was established as a result of the 1991 Gulf War, following the Israelis’ fear of the Palestinian reaction to the war (Tawil-Souri, 2009; Tawil-Souri, 2010). The development of a more serious system of closures followed Oslo II’s discussions at Taba in 1995 when the West Bank was officially divided into sections under Israeli control and Palestinian control. In response to this new division of territory, Israel enforced new restrictions on Palestinian movement to protect Israeli settlements within the West Bank. Israel further tightened control over movement in the West Bank after September 2000, when the second Intifada started resulting in the establishment of a network of hundreds of checkpoints and road barriers (Giacaman et al., 2007a, 2009; Mataria et al., 2009). Finally, although Israel withdrew its settlers from the Gaza Strip in 2005, by the end of the second Intifada in 2005, a comprehensive network of checkpoints had already emerged, and on November 15, 2005, an Agreement on Movement and Access (AMA) was signed, in which Israel assured the facility of movement of people and goods within the West Bank.

Furthermore, since 2005 the concept and the construction of the checkpoints have undergone fundamental changes by the Israeli government, which transformed checkpoints into terminals, in an attempt to create an appearance of legal and regular border crossings (Goldner and Scharf, 2012a). The structural changes included both architectural and administrative forms (e.g., changing the checkpoints into terminals, operating the checkpoints by both private “security” companies and IDF soldiers), while the conceptual changes have mainly been semantic and are part of the Israeli government’s ongoing attempt to redefine “terminals” as legitimate border-crossing points (Mansbach, 2009; Amir, 2013).

To date, studies on checkpoints are relatively rare, critical in nature, and adopt sociological and political points of view. They have described the construction and the functions of the checkpoints as an economic and commercial arena (Amir, 2013; McNeely et al., 2014), the mechanisms through which violence are justified (Kotef and Amir, 2011), or the Palestinians’ political opinions toward peace processes (Longo et al., 2014). To the best of our knowledge, only one study has discussed the psychological impact of border-crossing experiences. However, that study was conducted with Palestinians from Syria and described the psychological effects of crossing the border between Lebanon and Syria. The study demonstrated participants’ feelings of forbidden life and social death (Heide-Jørgensen, 2014). In the absence of research exploring the psychological influences of repeatedly passing through a checkpoint, using the framework of Palestinians living under conditions of chronic conflict, the current study endeavored to examine the mental experience of Palestinian adults who pass daily at the Qalandia checkpoint.

## Materials and Methods

### Participants

Twenty adult participants (50% females) between the ages of 19 to 58 (*M*_age_ = 13.47, *SD* = 0.34) who cross the Qalandia checkpoint participated in the study. The participants were recruited using snowball sampling with two inclusion criteria: All participants had to be Palestinian in origin and live in or near the Qalandia refugee camp. Fifteen participants (75%) lived in Kufar Akab, an Arab neighborhood of Jerusalem, which is mostly located in the northern part of the city. The neighborhood is located near the Qalandia refugee camp, about 2 Km south of Ramallah and 8 Km north of the center of Jerusalem. Estimates regarding its number of residents range between 18,000 and 80,000. Seventy percent of its residents are in the municipal area of Jerusalem, and 30% are in the area under the responsibility of the Palestinian Authority. Four participants lived in the Shuafat refugee camp, a Palestinian refugee camp located in northeast Jerusalem. It is the only Palestinian refugee camp within the municipal boundaries of Jerusalem, with an estimated 8,000 to 20,000 residents. One participant lived in the Qalandia refugee camp.

Many people who live in Kufar Akab and the Qalandia refugee camp have two houses. One is a small house in East Jerusalem, whose address is registered with the Israeli Ministry of the Interior and grants them their rights as Palestinians with Israeli residency, while the second house is bigger and serves as a residence. This participant’s house was built in the Qalandia refugee camp but in area C, which is under Israeli supervision. Participants had to cross the Qalandia checkpoint on a daily basis and have Israeli residency (a blue identity citizenship card) that permitted them to cross the checkpoint. We excluded from the study Palestinians from the oPt because they do not have Israeli permission to regularly cross the checkpoints on a daily basis. All the participants were Muslims. Fifty-five percent of the participants were employed, and 25% were students; 50% were single, 35% were married with children, and the remaining 15% were divorced.

### Setting

The study was conducted at the Qalandia checkpoint. The Qalandia checkpoint is the largest and most frequented passageway for Palestinians who need to cross between the occupied West Bank and East Jerusalem for work, school, getting to a hospital, or visiting relatives. Around 26,000 Palestinians pass through Qalandia daily on foot, by bus, or by car and they must follow Israeli authorities’ instructions. Queues form at the checkpoint from before the break of dawn. Traffic conditions at the checkpoint vary from hour to hour. Sometimes the authority checks are quick, but at other times there is lengthy questioning or delays. Thus it is impossible to predict how long it will take to pass the checkpoint. However, during rush hours (mainly between 4:00 to 5:00 am and between 5:30 to 7:30 pm) persons crossing take into consideration that thousands of people have to pass together and that crossing the checkpoint will take at least an hour and a half and sometimes even 3 h. There are also times that the checkpoint is unexpectedly closed and then crossing is not permitted at all.

### Procedures

Following receiving ethical approval for the study from the Committee to Evaluate Human Subject Research of the Faculty of Health Sciences and Social Welfare of the University of Haifa, the first author, who is an experienced music therapist, met the participants separately for an unlimited amount of time in their home. The researcher explained the aim of the study and obtained participants’ written consent. During this session, both verbal and non-verbal techniques were used to capture participants’ experiences of the checkpoint crossing. First, a semi-structured interview was conducted in Arabic and recorded (and later transcribed and translated into English). Next, participants were asked to engage in the drawing task. Participants were told that they were entitled to stop the interview or the drawing task at any point. The artworks and recorded interviews were stored safely under a code number with no identifying details.

Due to the sensitive nature of this study, participants were given the personal contact information of the researchers, who are all experienced therapists. The participants also had access to public support centers if they experienced any distress or if any questions arose. Throughout the process of writing and consolidating this research, every effort was made to ensure that the life stories and artworks of the participants were presented respectfully, without changing any of the facts or the essence of the content.

### Instruments

#### The Semi-Structured in-Depth Interview

The purpose of the semi-structured interviews was to gather the in-depth meaning of participants’ passing the Qalandia checkpoint on a daily basis. The interviews were based on a questionnaire guide, which was composed of a list of open-ended questions aimed to elicit descriptions of the participants’ experiences and how they influenced their lives. It included questions such as: “How is it for you to pass through checkpoints?” “Please give a metaphor to illustrate this experience.” “Please describe the most significant experience that you remember regarding passing through the checkpoint. Explain its significance to you.” “In what way is passing through the checkpoints influenced your life?”

#### The Drawing Task

As internal distress is, in many cases, covert, this study, as have many others, used drawings by the participants to provide an additional channel of learning about their inner experience (e.g., Lev-Wiesel and Liraz, 2007; Goldner and Scharf, 2012a). Participants received a blank sheet of paper, size A4 (21 cm × 27.9 cm) and a package of crayons with twelve colors, and were requested to draw an image that reflected their experience with the checkpoint. The drawings could portray their experience abstractly or figuratively. The use of drawings is considered a useful tool among scholars and clinicians, as it allows the expression of hidden or repressed thoughts and feelings in a relatively rapid and simple way by passing the censorship defensive mechanism. In particular, since traumatic events are often recalled in a fragmented, dissociated, and non-verbal manner the use of drawings can provide a unique approach for research by allowing the trauma to reveal itself in a visual form (Malchiodi, 1998; Rubin, 2005; Gantt and Mills, 2009). The assumption is that by using colors, shapes, and motifs in visual content, the unconscious can be expressed, adding layers of meaning to the stated verbal content (Betts, 2006; Ellis, 2007; Lev-Wiesel and Liraz, 2007). The use of an integrated approach that involves the use of both visual and word-based research methods offers a way of exploring both the multiplicity and complexity that is the basis of much social research interested in human experience (Guillemin, 2004; Betts, 2006). In the current study, we focused on the inner psychological meaning of daily crossing the Qalandia checkpoint and looked for additional channels of expression.

### The Analysis

Interpretative phenomenological analysis (Smith et al., 2009) was deployed while analyzing the interviews to achieve depth insight regarding crossers’ perceptions and meaning construction prior to the establishment of a theoretical interpretation. Each interview was looked at separately, and the analysis by the three authors started with an intensive reading and rereading of the interview transcripts and notes so the researchers were familiarized with the contents. Any significant and potential meanings were noted and written down. A preliminary list of initial categories was created from which a second list connecting the themes was created, illustrating the clustering of these themes into a smaller number of higher-order ones that were checked against participants’ phrases to ensure they supported the connections made. A table of themes was then developed by giving a name to the clusters of themes to form superordinate themes.

The analysis of the drawings was conducted by the first and the second authors, who are experienced creative art therapists. The second author is also an experienced researcher in the field and specialized in using and developing art-based assessments. The analysis of the drawings was conducted using a phenomenological analysis approach in *art therapy* (Betensky, 1995; Hazut, 2014). The analysis focuses on the particular way in which individuals perceive their lives by identifying common repetitive pictorial phenomena, which refer to certain features of the pictorial art such as lines, colors, shapes, images, textures, contrasts, massive areas, space, and the relations between them (Simon, 2001; Huss et al., 2012; Thyme et al., 2013). Next after, the information that emerged from the analysis of the various drawings was gathered into broad, global, and aggregate pictorial phenomena based on the organization, aggregation of signs in the drawings, and general impression. Namely, we search for similar pictorial styles in various drawings to understand certain experiences. In the end, interpretive psychological theories that assess mental functioning through compositional and stylistic elements were used to produce meaning from the phenomena (Huss et al., 2012).

To ensure inter-coder reliability, both regarding the drawings and the interviews, we performed thematic content analysis or pictorial phenomenological analysis separately. Subsequently, we compared individual analyses, discussed disagreements, and looked for conformity regarding theme and pictorial phenomenon content and the interpretation of meaning. We rejected themes considered to make a minor contribution to understanding the examined phenomenon to achieve parsimony.

Finally, the research team includes both Palestinian and Israeli researchers who are interested in the psychological impacts of various trauma situations. The use of a mixed research team group and the application of a reflexive approach and transferability to detect the researchers’ perspectives, positions, values, and beliefs (Barry et al., 1999; Malterud, 2001) may have assisted in minimizing any hidden agendas or preconceptions. On a personal note, the Israeli authors felt compelled to find out how Palestinians experience the checkpoints, believing that the two populations are destined to live side by side forever in this region, so attaining a broader understanding of the Palestinians’ suffering through narratives and drawings is necessary. The Palestinian researcher grew up in East Jerusalem and used to cross the checkpoints on a daily basis. Today, as a Palestinian citizen of Israel, she usually visits her family and crosses the checkpoints once a month with her family. She wished to learn about the suffering of the Palestinians she meets and its relation to her identity as a Palestinian.

## Findings and Discussion

### The Interviews

The analysis of the interviews yielded three central themes. The first theme described deep feelings of distress and desperation as manifested explicitly and implicitly. This theme was comprised of subthemes of feelings of humiliation and dehumanization, non-existence, rage, and pessimism and hopelessness. The second theme focused on the participants’ coping strategies, concentrating on aspects of habituation and dissociation, while the third theme described the social fragmentation of the Palestinians’ solidarity (for a summary of the interviews’ themes and drawings’ phenomena, see **Table 1**).

**Table 1:** Verbal themes, subthemes, and pictorial phenomenon.

Interviews		Drawings
**Themes**	**Subthemes**	**Phenomenon**
(1) Deep feelings of distress and desperation		(1) Squared restricted drawings
	(1.1) Feelings of humiliation and dehumanization	(2) The use of multiple black tiny objects
	(1.2) Feelings of non-existence, danger, and dying	(3) The use of split drawings
	(1.3) Feelings of rage	
	(1.4) Feelings of pessimism and helplessness	
(2) Coping styles		
(3) Fragmentation of the palestinian solidarity		


#### Deep Feelings of Distress and Desperation

##### Feelings of humiliation and dehumanization

Two central feelings that participants expressed were humiliation and dehumanization. Feelings of dehumanization are clear from the interviews: participants described themselves as “thrown objects” that were required to be inspected. They reported having to obey any given order or face additional inspections, delays, or severe punishment. “The soldiers don’t see that we are human beings crossing the checkpoint.” “If you blow your horn or even show that you are in a hurry, they [the soldiers] will catch you … He [the soldier] will yell at you, ‘Come! Pull over!’ … and they delay you.” (Sh., male, 33, driver) Other checkpoint crossers felt that the dehumanization went much further. They remarked that to follow the soldiers’ orders, they were forced into adopting abnormal, humiliating behaviors, and ultimately they felt like animals. Furthermore, participants constantly used the word “human” during their interviews to emphasize their loss of human identity, namely the sum of the components that define the essence of being human, such as meaning making, adopting values and moral behavior, and pursuing life goals (Agustin, 2009; Albarello and Rubini, 2012; Génova and Quintanilla Navarro, 2018). For example, participants said, “They treat us like animals. When I am obliged to make my daughter poop in the car! It means we have no dignity.” (M., female, 34, engineer) “We feel like we are not human beings.” (A., male, 25, cook; H., male, 27, worker). “I feel I’m an abnormal human being.” (Sh., female, 37, teacher).

While feelings of humiliation are apparent from the above descriptions of dehumanization, the sense of humiliation was further evident in participants’ descriptions of their relationships with the soldiers. Many participants compared their relationships with the soldiers to the relationship between detainee and jailer, in which the soldiers represent commanders in charge of the prisoners and they, the checkpoint crossers, represent the detainees. Some of the participants used the words “cage,” “prison,” and “jail” to describe the denial of dignity, which conveyed checkpoint crossers’ feeling of being psychologically trapped. They explained how the soldiers’ orders had to be obeyed to ensure crossing the checkpoint. They compared themselves to prisoners, criminals, and guilty suspects, which lent an additional sense of danger to the experience. They said: “We are imprisoned here.” (R., male, 23, social worker) “It’s just the same as a prison … they inspect you every day.” (S., male, 46, driver). “If you sit quietly in your car you will pass.” (Sh., male, 33, driver) “Stop!’ [says the soldier] We stop. ‘Go!’ [says the soldier] We go.” “When I cross the checkpoint, I’m a suspect. I feel like I am accused of something, and I don’t know what it is.” (A., female, 54, school counselor). “While I’m standing in line waiting, he [he soldier] comes to me and orders, “get out of the car!”… He just didn’t like the way I looked. He told me to park my car on the right side of the lane! 15 min passed, 30 min passed, then he came to inspect the car as if he was searching for something. He brought the dogs for an additional inspection, he made the dogs circle around the car, nothing! There was nothing, he just intended to delay me.” (M., male, 53, driver).

Another humiliating aspect of the checkpoint crossers’ relationship with the soldiers was the setting up of the soldier as “superior” and the passenger as “inferior.” Participants described the soldiers’ behavior as sometimes rude, lacking empathy, apathetic, and inhuman. All the participants in the study agreed that the soldiers’ mood determined whether that day’s crossing would be easy or difficult or whether the inspection would be conducted in a respectful or humiliating manner. For example, checkpoint crossers said: “He sits in a high chair, putting his legs through his window, and we are forced to see and tolerate his arrogance.” (S., female, 19, student) “They [the soldiers] are bossy all day and night and think we count for nothing.” (N., female, 19, student) “He [the soldier] brought the dogs for an additional inspection; he made the dogs circle around the car, nothing! There was nothing; he just intended to delay me.” (S., male, 46, driver) Others believe that the soldiers deliberately use unjustified power designed by the Israeli government to amuse the soldiers or degrade the checkpoint crossers. “They bring the worst inhuman soldiers to the checkpoints to humiliate us.” “Usually the soldiers use us to make fun of us.” (I., female, 54, teacher).

In describing their relationship with the soldiers, participants also expressed feelings of being tormented and assaulted, by using the words *Marmata*, *Atta’tas*, and *Sammet Baddan*. Marmata means “being held by the hair and pulled sharply from one side to another.” Atta’tas denotes “destroying someone and making them feel miserable and ruined,” while the expression Sammet Baddan means “the insertion of poison or toxic material inside the body.” They described figuratively the torture and the aggression that the body and soul must confront. For instance, participants said: “I go through Marmata every time I cross the checkpoint.” (S., female, 19, student; S., female, 24, cleaner; H., male, 26, chef’s assistant).

The emotion of humiliation has been recognized as a central emotion among Palestinians in the oPt. This emotion has been defined as an internal negative experience of the victim as a result of being intentionally unjustly treated and degraded (Giacaman et al., 2004). It can be experienced on an individual as well as on a societal level and thus can yield massive negative effects. Furthermore, researchers have suggested that humiliation is a central deliberate tactic used by dominant groups aiming to violate dignity and basic human rights as a form of political control and that it has profound, negative consequences (Lindner, 2001; Giacaman et al., 2004; Giacaman et al., 2007c). It seems that this description corresponds to the experience of the study participants, who experienced daily dehumanizing treatment by soldiers. According to the participants, the soldiers took advantage of their power by treating the checkpoint crossers as their masters or jailers and using unjustified power deliberately to frighten and degrade the travelers to the extent that they felt robbed of their dignity and humanness.

##### Feelings of non-existence, danger, and dying

Another major theme that was expressed in the interviews was the feeling of death and non-existence. Checkpoint crossers routinely used the metaphors of “dying,” “going to the grave,” and “annihilating” to describe the experience of death they faced at the checkpoint. “Checkpoints are death!” (R., male, 58, worker) “When I approach the checkpoint, it feels like I am going to the grave.” (A., male, 25, cook).

Over and over, participants described the feelings of nihility, worthlessness, invisibility, and irrelevance. They stated: “The soldiers don’t look at us in the first place, they don’t see that we are human beings crossing the checkpoint.” (S., female, 19, student) Checkpoint crossers felt that their lives had no meaning or value? at the checkpoint. Some participants, based on their own experiences and others that they witnessed, talked about the danger of losing one’s life at the checkpoint by making the slightest mistake. They underscored the need to be constantly alert to any signal of imminent threat while standing in line. For instance, a few participants stated: “You might get shot if you make a slight mistake [e.g., move or speak when not told not to do so], that’s why we must be careful and take extra precautions.” (R., male, 23, social worker; Sh., male, 33, driver; M., male, 20, student) “When a person moves forward in line, if he doesn’t notice any gesture from the soldier, he might get shot, it happened to me, but I didn’t get hurt, thank God.” (M., male, 53, driver) “When the terminal is empty and someone is crossing alone, a mistake may occur, especially if this is their first time, if they missed the right route, or if they are used to crossing by car and don’t know the rules for those who cross by foot. You might get shot if you make a mistake, that’s why we must be careful and take extra precautions.” (M., male, 20, student) This sequence of idioms aligns with previous findings regarding Palestinian human insecurity in the oPt, comprised of constant feelings of concern and fears regarding home displacement, loss of home, economic welfare, worries about the future, and safety of life (Qouta et al., 2008; Mataria et al., 2009; McNeely et al., 2014).

This situation they experienced as unbearable raised a series of questions for the checkpoint crossers as to what was considered normal or abnormal. They contemplated the normality of their reality and longed for the normal life that they had lost. Seeking to better understand their situation from a logical perspective, the participants raised questions regarding the ability of normal persons to endure the amount of suffering that they endured. They questioned whether people in other places would tolerate this abnormality: “I ask myself daily, why? Why us? Why are we living like this?” (S., female, 26, psychologist). “Do you think those who live in Tel Aviv would tolerate living in the fear, stress and daily pressure to reach their university?… Why are we, Palestinians, forced to live like this and the rest of the world doesn’t?” (S., female, 19, student).

Feelings of non-existence and being destroyed, devastated, and psychologically drained have been described as prominent fears of the oPt Palestinian, serving as risk factors in demolishing Palestinians’ well-being (McNeely et al., 2014). We suggest that the intensity of the feelings of non-existence, danger, and dying reported here can be understood in light of the impossible and exhausting demands upon the Palestinians to live their lives in a way that normalizes the abnormality while keeping them on the threshold of catastrophe and disaster. The experience of the threshold, because of its instability and liminal nature, hampers the perception of certainty of time and space radically (Handel, 2009). Normalizing the abnormality, it undermines checkpoint crossers’ sense of normality and existence.

##### Feelings of rage

Some of the participants described feelings of rage, literally and metaphorically using embodied metaphors. These descriptions can be understood as a natural human impulse in response to the frustration and the experienced humiliation (Mohamad, 2007). For instance, some participants used explicit expressions or anger and rage while crossing, saying: “While crossing the checkpoint I feel enraged.” (Z., female, 48, teacher) “If I were able to smash him, I would.” (I., female, 54, teacher) Other participants widely used the word “blood” and its transformation to “boiling,” “burnt,” or “poisoned” to illustrate their level of rage and the agony they experienced. “I feel my blood boil. I boil on the inside.” (S., female, 24, cleaner; Z., female, 48, teacher; Sh., male, 33, driver) Some of the participants used the metaphor of insertion of poison into the body to represent the pathological and the toxic situation that invades and spreads out into their lives and imposes psychological danger. They stated: “The congested traffic that we face daily poisons our body.” (M., female, 34, engineer) “We have given a name to the checkpoint; it is the ‘blood poisoning’! … We ask each other, where are you going? We answer, to the blood poisoning.” (S., male, 46, driver).

Embodiment-metaphorical language to describe anger is a universal? form to express anger (Maalej, 2004). In this case, aspects of the language are structured by the features of our bodies and the functioning of our bodies in everyday life (Goschler, 2005). It appears, in the present study, that this kind of language helps the crossers to communicate their life-threatening and exhausting experience that seems beyond their understanding and their attempt to escape from the feelings of weakness and helplessness. The feeling of rage can be seen as a normal counterreaction of adjustment and strategy for preserving a sense of self-coherency that blurs in the relationship with the offender, control, and self-worth (Van Velsor and Cox, 2001). In this respect, rage may be seen as a natural response to the offender for the unjust treatment, in order to achieve a certain justice and subjective well-being (Tripp et al., 2002).

##### Feelings of pessimism and helplessness

Feelings of pessimism helplessness was pervasive throughout the checkpoint crossers’ answers, with the interviewees expressing physical tiredness and mental exhaustion. They felt depleted of energy, stating: “I become pessimistic when I reach the checkpoint … the checkpoint has not only affected my life, it has executed me.” (A., male, 25, cook) “Damn this life! Why? I hate work and the day I go to work.” (Sh., female, 37, teacher) “I go to work in order to give of my positive energy, but after I cross the checkpoint, all that energy is gone and substituted with negative energy that accompanies me all day long. I am tired of going through every day.” (S., female, 19, student).

The feelings pf pessimism and helplessness were also evidenced by the checkpoint crossers’ perception of time. Participants maintained a fatalistic, helpless, and hopeless attitude about the present. Their description of their present life was narrowed to the survival of the checkpoint physical conditions, such as opening hours and the length of waiting time, emphasizing the loss of time and money, with sentences such as “To leave at 5 means I gained 1 h, it’s like winning gold.” “After I cross it I feel I got released from all of the pressure I went through while crossing.” (R., male, 58, worker).

Reference to the future was almost absent from the interviews, while feelings of helplessness dominated the conversations. Participants perceived their reality as a fatal destiny that one could not change (although at times seeming to find support in their Muslim faith). “I have nothing in my hand that I can do or change, and the only complaint is to God.” (S., male, 46, driver) “The situation at the checkpoint is hopeless.” (H., male, 26, chef’s assistant) “I’m fatigued. There is no solution.” (S., female, 26, cleaner) By contrast, reference to the past was mainly warm, sentimental, and nostalgic. Participants longed for the time when they moved freely from one place to another, saying, “There were days when I used to finish my working day at 4 or 5 pm, then come home and take all the family and go to Tel Aviv; there were no obstacles on the way.” (R., male, 58, worker).

In addition, during the course of the interviews, participants described having no control over their time. The checkpoint was perceived as a significant obstacle in their lives, as they could not make any plans without bearing in mind their limited ability to predict the events at the checkpoint. To illustrate, most participants used the words “it depends” as a key phrase to demonstrate their state of being conditioned and unsure about their daily routines. “A huge part of my life has become a complete mess because I can’t plan anything!” (A., female, 54, school counselor) As a result, they feel deprived and robbed of their basic rights and needs, such as the right to control their time and consequently their life. “I feel a portion of my time and life is lost, I lose hours and precious moments.” “I feel someone is robbing me. I feel they are stealing part of my life … my entire being.” (Z., female, 48, teacher).

Although this pessimistic, hopeless cognitive style is akin to the phenomenon of learned helplessness (Seligman, 1972, 1975), which refers to the disposition of individuals to remain passive and accept adverse situations after exposure to repeatedly painful or aversive stimuli (Seligman, 1975), these feelings cannot be seen as typical symptoms of mental despair but rather as an expression of social suffering that is associated with the injustice and violence of the occupation. This claim is consistent with criticism more broadly that diagnostic categorization of disorders and pathology is applicable in normative situations of sadness and suffering (McNeely et al., 2014).

#### Coping Styles

Prior studies in armed conflict areas identify both adaptive and non-adaptive coping styles to preserve a certain degree of well-being. Whereas the adaptive coping style includes strategies of seeking humanitarian help, enhancing literacy and education, increasing political involvement (Punamäki, 1990), meaning making, and seeking spiritual and social support (Kulogğu-Karsli, 2013; Say, 2017), the less adaptive coping styles were comprised of avoidance, denial, and emotion-focused coping strategies (Punamäki et al., 2008; Mousa Thabet and Vostanis, 2017). In our study, in response to the extreme difficulties and to better acclimate themselves to their daily challenges, participants responded in various ways that consisted of cognitive and behavioral coping strategies of habituation, including tactics of adaptation and acceptance, as well as mental defensive coping mechanisms of dissociation, manifested in disengagement and complete ignoring of the soldiers. Cognitively, participants reported accepting the situation of the checkpoint and referring to it as “normal.” They stated: “The checkpoint has become routine in our everyday life, and we deal with it automatically.” (Sh., female, 37, teacher) “I have to tolerate this situation because it’s compulsory, and we can’t do anything about it.” (S., female, 26, psychologist) Behaviorally, the dominant response among the coping styles was avoidance: unless it was essential, participants avoided entering the checkpoint and interacting with the soldiers. They stated: “I think a thousand times before I go to visit relatives.” (A., female, 54, school counselor) “I’m not willing to reach the checkpoint unless I’m obliged; otherwise, I stay home.” (A., male, 26, cook) Other coping styles included appropriating extra time for the crossing, using social media to assess the situation at the checkpoint, and learning the soldiers’ behaviors before the inspection, to better predict and achieve a sense of control over the situation.

The intensity of negative and distressing feelings that were caused by the daily crossing compelled the participants to dissociate themselves and to disengage emotionally from the soldiers, even refusing to recognize them. “I have quit confronting them [the soldiers], I don’t have time to argue with her [the soldier], I have to pass.” (I., female, 54, teacher) “In a period of time, I used to have a huge amount of rage whenever I approached the checkpoint, as if I was waiting for a slight click to fight with them, but now I have been exposed to a lot of things by them [the soldiers] so I decided to nullify them. Now, I can’t recall any face of a soldier, I cross each day, but I don’t distinguish any of them, I don’t remember them.” (Z., female, 48, teacher) “If I keep recalling their faces, my life will be affected, so to keep my psychological state comfortable, I decided to cancel their faces.” (A., female, 54, school counselor).

These defensive coping strategies of habituation and dissociation are aimed to bypass and ease the checkpoint crossers’ emotional pain, fear, self-harm, exposure to violence, and humiliation (Stellrecht et al., 2006). They are typical to trauma survivors in the context of armed conflicts (Peltonen and Punamäki, 2010), adult refugees (Finklestein and Solomon, 2009), and war veterans (Kashdan et al., 2010) and are similar to those described in studies that have investigated the effects of collective traumatic events, such as civil war, war conflicts, and disaster situations (Abramowitz, 2005; Somasundaram, 2007, 2010). Specifically, in the case of habituation, the repeated exposure to pain and violence may cause acclimatization to the violence that makes it less aversive and in some cases may lead to aggressive behaviors toward the self or others (Guerra et al., 2003; Lev-Wiesel, 2005; Joiner et al., 2007), while in of dissociation, in attempt to disengage from traumatic memories the traumatic experience is not fully integrated into one’s existing cognitive schemas (Lemos-Miller and Kearney, 2006; Shelef et al., 2014).

It appears that in the present study that these reactions serve as adaptive responses in the short term as they serve as a protective function by reducing conscious awareness of the overwhelming emotions during the crossing. Nevertheless, it should be noted that the dichotomy into adaptive and non-adaptive coping styles is especially appropriate for Western society and clinical situation and does not take into account massive situations of collective trauma.

#### Fragmentation of the Palestinians’ Solidarity

Checkpoint crossers reported the adoption of aggressive tactics toward members of their community and mourned the loss of their social solidarity and posited that the checkpoint dismantles the cohesion of their community by undermining its ability to act as a group (Somasundaram, 2007). In particular, the waiting time at the checkpoint led people to adopt an offensive manner toward each other. They described the waiting area as an area of anomie and chaos, lacking rules and order, characterized by a wild competition to try to cross more rapidly. Participants said: “The craftiest and experienced person [ironically], is the one who comes in front of other people, skips all the traffic congestion and crowded people, and arrives first at the line.” (Sh., male, 33, driver) “I might upset five people to gain 5 min.” (S., male, 46, driver).

Even though many participants shared and recollected the same humiliation, pain, and suffering, they did not feel caring and sympathy for others’ pain while waiting in line. Emphasizing the change in the nature of people and the transformation from victims into aggressors, they asserted, “We have been transformed into a people who violate each other.” (I., female, 54, teacher) “We are being oppressed by both the Arabs and the Israelis.” (S., male, 26, driver) “When you ask those crossing the checkpoint by car to give you a ride some people turn their faces and look toward you arrogantly.” (Sh., female, 37, teacher).

The fragmentation of solidarity was also revealed in participants’ descriptions of checkpoint crossers’ treatment of Palestinian children, who sell goods at the checkpoints. On the one hand, people expressed sorrow for the children who were required to earn even a little bit of money. On the other hand, they described their irritation at these children, who urge drivers and pedestrians to buy their goods. One of the checkpoint crossers asserted, “This generated a gap between how people would normally handle or treat children and how they treat children at the checkpoint. Some people tend to reject talking to them or simply ignore them, which is something they don’t usually do when talking to children anywhere else.” (Z., female,48, teacher).

The fragmentation of social connectedness and the social fabric has been suggested as a central aspect of social suffering, war zones, and conflict areas (Erikson, 1978; Abramowitz, 2005). Communities trapped in trauma cycles often are breeding grounds for severe social problems and chronic violence. In particular, traumatized communities are vulnerable to exploitation, radicalization, societal fragmentation and polarization, which are sources of tribal violence (Riedel, 2017). Several explanations have been suggested to better understand participants’ offensive behavior toward each other. Fanon’s theory (Fanon, 1963) on politics and violence may be useful. The theory proposes that abnormal aggressive behavior exhibited by colonized individuals reflects a defensive operation to master passively endured trauma through active repetition to avoid re-experiencing feelings of helplessness, terror, and anxiety (Lewis and Bucher, 1992; Garland, 1993). On a psychological level, the aggressive behavior can be understood as a replication of the abusive behavior as part of the habituation process and identification with the aggressor (Guerra et al., 2003). Another explanation has been proposed by Riedel (Riedel, 2009, 2017). She suggests that in collective trauma situations, individuals move on an aggression-depression axis in an attempt to deal with harsh reality. In some cases, the aggression is directed inward, while in others, it is directed outward, causing the fragmentation of the society and diminishing its solidarity. Thus, categories such as perpetrator and victim no longer apply.

### The Drawings

One of the strengths of the present study is the inclusion of a projective technique to learn about the participants’ inner experience through identifying several pictorial phenomena in the participants’ drawings, which enabled another layer of the experience of the participants’ helplessness and vulnerability to be revealed. Previous works have examined the associations between artistic expressions and trauma (e.g., Lev-Wiesel, 2005; Avrahami, 2006; Huss et al., 2012), with the assumption that the encoding of traumatic memories may occur via a photographic visual process (Johnson, 1987). Our analysis yielded three pictorial phenomena that emerged from the participants’ drawings: squared restricted drawings, the use of multiple black tiny objects, and the use of split drawings in red–black color combinations. Generally, the drawings validated participants’ verbal descriptions regarding their experience of helplessness, humiliation, rage, and internal distress.

#### Squared Restricted Drawings

One fundamental phenomenon of drawings that emerged from the artworks of the participants was that of squared restricted drawings, which appeared in 7 of the 20 drawings. It was manifested in the depiction of squares, borders, and barriers. These squares were often emphasized or doubled. In some cases, within the squares, different types of objects (people, balls, and bombs) were trapped in a square and could not get out? Furthermore, the color scale of these drawings was relatively limited, and there was almost no referral to the surroundings.

**FIGURE 1:**
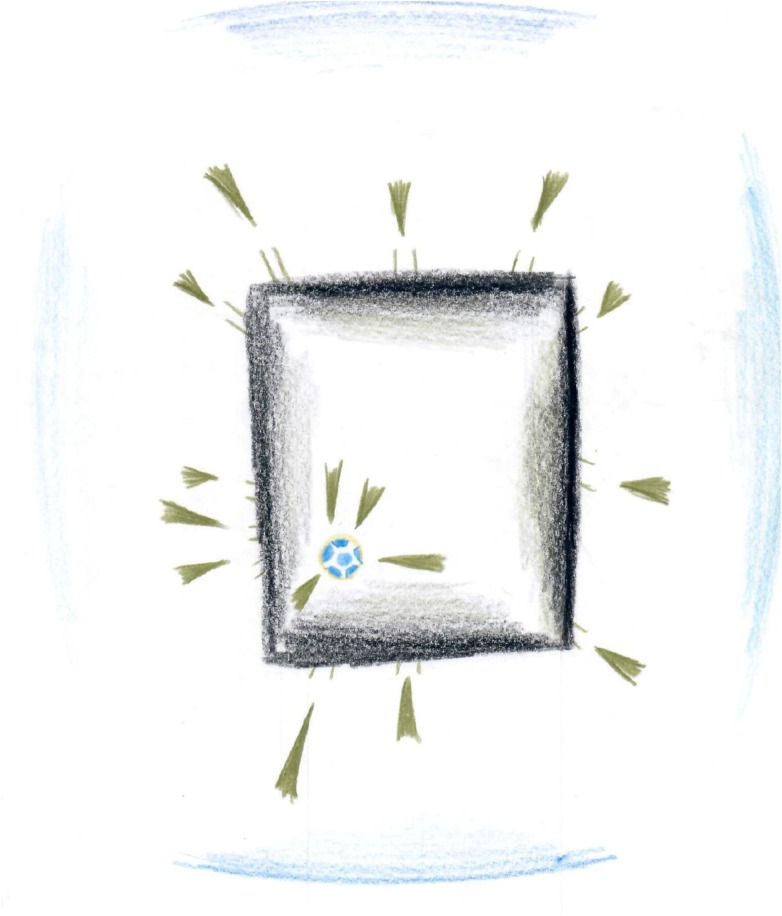
Squared restricted drawings.

**FIGURE 2:**
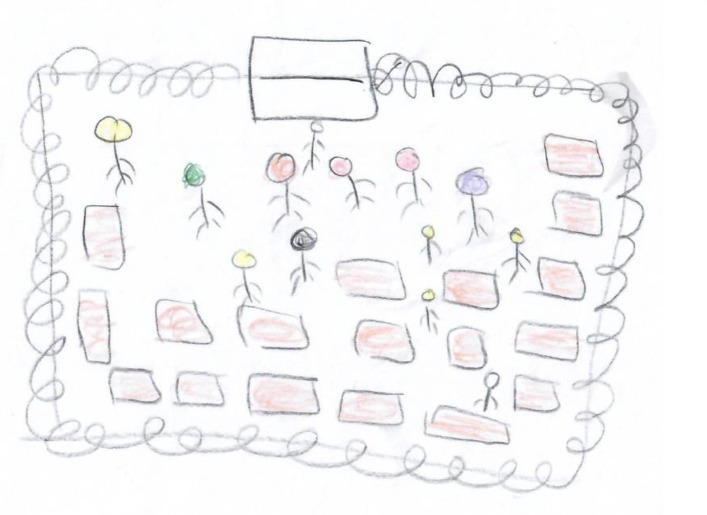
Squared restricted drawings.

The overall impression was of imprisoning, lack of a way out, and helplessness instilling an overall feeling of restraint (see **Figures 1, 2**). This feeling of “no way out” was also revealed in participants’ descriptions of their drawings. For instance, in **Figure 1**, a black square trapped a ball. The participant stated: “I drew a ball, the ball represents me. I drew a ball because I feel they play with us just like a ball.” (R., male, 23, social worker) Concerning **Figure 2**, the participant said: “I drew a prison with a crowd of people and they only have one place to leave it.” (S., female, 19, student). This impression aligns with the description of feelings of pessimism and helplessness which was reveled in participants’ interviews. In addition, this brings into line with previous studies that suggested that the use of squares serves as an expression of internal distress and a negative emotional state. For instance, in a study that compared emotional content drawn on circle versus square formats, the researchers found happier and more positive emotional content expressed within the circle format, compared to angry or hostile feelings revealed on the square format (Slegelis, 1987).

#### The Use of Multiple Black Tiny Objects

**FIGURE 3:**
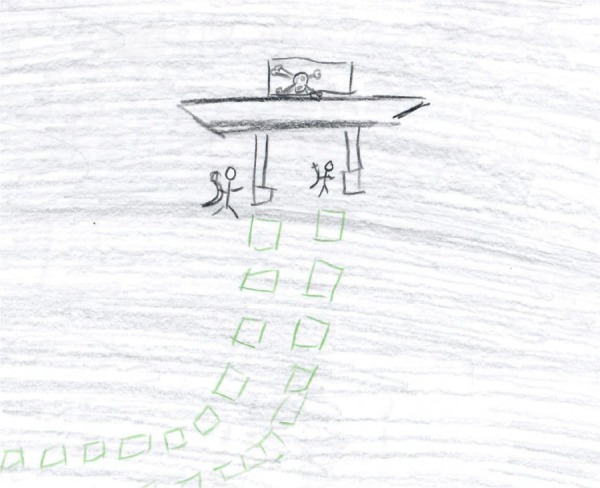
The use of multiple black tiny objects.

**FIGURE 4:**
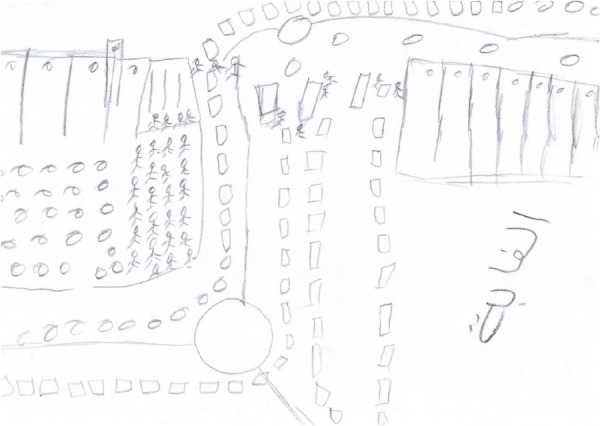
The use of multiple black tiny objects.

The second phenomenon, which was revealed in twelve of the drawings, was the use of multiple tiny objects that concretely depicted the checkpoint. These drawings included multiple, crowded, anonymous, and primitive small figures-multiple tiny squares or cars organized in lines that crowded the edges of the page. The dominant color of these drawings was black (see **Figures 3, 4**). In some cases, the weapons of the soldiers were included. Despite the number of objects, the tininess of the objects and the anonymity of the figures generated an overall impression of humiliation, vulnerability, danger, and loneliness, maybe to reflect the helplessness of the society, validating participants’ narratives regarding their perceived humiliation and insecurity. Moreover, the density of the objects produced a sense of suffocation and dying as also reveled in participants’ stories (see **Figures 3, 4**). The sense of vulnerability also manifested in participants’ narratives. For instance, S., 33, a truck driver who drew **Figure 3**, described his drawing: “I feel that there are persons whom their job is to take our souls away and they anticipate seeing me having a stroke.” H., 26, a sheaf assistant who drew **Figure 4**, wrote on his drawing the word “Jungle,” which denotes a place of survival, a place where only the strongest survive.

The use of tiny and anonymous figures or objects may reflect participants’ inner sense of vulnerability, incompetence, and non-existence. It is precisely the large number of similar objects created as if on a production line and the attempt to create order that reflects the participants’ sense of helplessness. The use of tiny figures has been found in studies of children’s drawings, in which the tiny figures mirror the child’s powerlessness, vulnerability, and anxiety (Carroll and Ryan-Wenger, 1999; Saneei et al., 2011; Goldner and Scharf, 2012b). Moreover, researchers have suggested that perseveration in drawings usually indicates a lesser degree of psychological well-being (Slegelis, 1987). Furthermore, multiplicity of figures in drawings were found to be used by survivors of childhood sexual abuse, reflecting their sense of inner fragmentation (Lev-Wiesel, 2005).

Moreover, these drawings were mostly depicted in the color black. Researchers have suggested that people in different emotional states choose and interact with colors in different ways (Lev-Wiesel and Dapna-Tekoha, 2000; Withrow, 2004). Studies have indicated that while emotionally well-adjusted individuals respond to color openly, people who are more emotionally distressed eschew color when possible, using limited color scales (Wadeson, 1971; Gantt and Tabone, 1998). In particular, black as a symbol represents primal ancient darkness, the blurred, and the unknown (Eisenbach et al., 2015). In addition, there is a broad consensus across cultures that black as a symbol represents death, mourning, sorrow, sadness, evil, grief, or mortification (Adams and Osgood, 1973; Cooper, 1978). Thus, in the present study, it seems to disclose, similar to participants’ narratives, the helplessness, sadness, and agony of the participants, as well as their lack of energy and passivity.

#### The Use of Split Drawings

**FIGURE 5:**
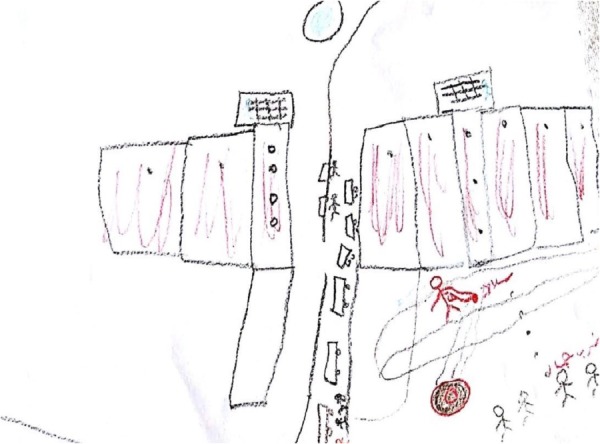
The use of split drawings.

**FIGURE 6:**
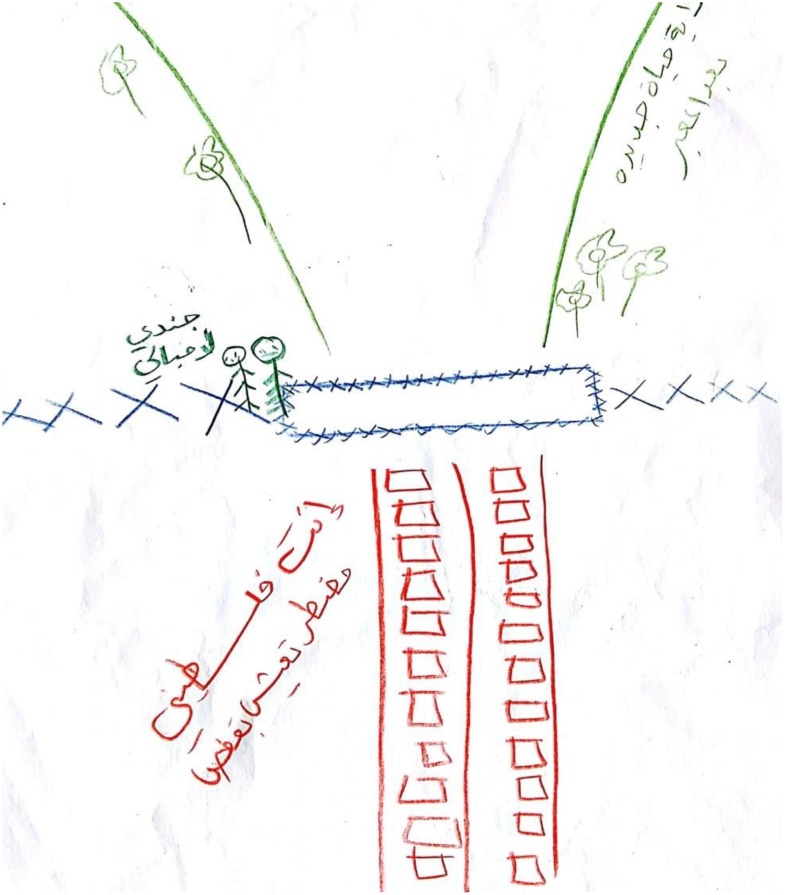
The use of split drawings.

The third phenomenon was the use of split drawings, which manifested in five drawings that were divided into two parts by barbed wire fences, barriers, roads, and X forms. Sometimes these drawings were drawn in a red–black color combination. In some drawings, the splitting line signifies the difference between the catastrophic present and the anticipated future. For instance, M., 34, an engineer, wrote on her drawing (see **Figures 5, 6**) “before crossing the checkpoint who is a Palestinian who is obliged to live in a cage.”

The split drawings may reflect participants’ sense of fragmentation and the coping mechanism of dissociation as was also emerged from participants’ verbal description. The use of split drawings was found among survivors of childhood sexual abuse and aligned with the defensive mechanism of dissociation (Riedel, 2017) that was also described by the participants. The red and black color combination creates drama and emphasizes the contrast. While black represents death, bereavement, and suffering, the color red signifies love, passion, energy, power, and fertility, as well as blood, pain, anger, struggle, and revenge (Adams and Osgood, 1973; Withrow, 2004).

In the present study, and comparable to the participants’ verbal content regarding their feelings of anger, red appears to represent crossers’ pain, blood, and injury in addition to their towering rage and their desire for revenge and achieving justice. Challenging the feelings of despair and depression, this combination may reflect the natural inclination of the psyche to move from destruction to life and into achieving a sense of control (Lev-Wiesel, 2005). Empirically, several studies involving “life-threatened children” (including adults who were sexually abused as children, earthquake victims, and children with leukemia) reveal a substantial prevalence of the colors red and black in their artwork (Cotton, 1985; Gregorian et al., 1996; Eisenbach et al., 2015).

### Conclusion

The current study sheds light on the psychological experience of Palestinians who were repeatedly passing through Qalandia checkpoint using findings gathered using both verbal and non-verbal research techniques. The analyses of the findings showed that enduring the abusive and threatening life experiences of the daily crossing generated immensely heightened psychological vulnerability, not just at the individual level, but at the community level, as part of continuous and extensive suffering. This was displayed in the adaptation of specific passive dissociative coping styles, fatalistic time perspectives, and fragmentation of the social fabric. Thus, we suggest referring to Palestinian distress not only on an individual level but considering their suffering as “social suffering” (Giacaman et al., 2004).

#### Clinical Implications

The study results reflect the psychological symptoms that Palestinians endure and social mechanisms that they employ in their daily journey crossing the Qalandia checkpoint. Being aware of the small effect size of trauma-focused interventions carried out in war-affected zones (Veronese and Barola, 2018) and the difficulties in isolating the suffering at the checkpoints from the suffering of everyday life, we suggest, with caution, developing programs to enhance Palestinian resilience, taking into account the checkpoint crossings. Specifically, we propose strengthening the Palestinian sense of solidarity and cohesion within the community as well as boosting their sense of control (Veronese and Barola, 2018) by relying less on defeatist coping styles. At the same time, the Israeli government should develop intervention programs for its soldiers to help them deal with the complexity of the situation, while addressing the Israeli security needs as well as the suffering of the Palestinians.

#### Limitations and Future Studies

Nevertheless, several limitations of the current study should be acknowledged. First, the findings of the present study reflect the experience of adult Palestinians crossing the Qalandia checkpoint, which involves aspects of the long-lasting political conflict. Thus, it is difficult to isolate the issue of the checkpoint from the daily, multilevel, and complex traumatic reality of Palestinians in oPt and social and cultural discrimination against Arabs living in Israel. Furthermore, this study is written from a psychological perspective rather than a sociopolitical one. Hence, future studies may wish to analyze the distress of the participants in light of local and global political processes, such as the failure of the peace process and the weakness of the Palestinian Authority. Also, by adopting a qualitative research design, the current study neither investigated the prevalence of the symptoms nor examined quantitatively the processes that generate these symptoms. Future quantitative studies should investigate how prevalent participants’ symptoms are and how they are revealed. Further studies also should examine children’s experiences regarding crossing the checkpoints.

## Author Contributions

NN conducted the interviews. NN, LG, and RL-W analyzed the interviews. NN and LG analyzed the drawings. All authors were involved in the writing of the article.

## Conflict of Interest Statement

The authors declare that the research was conducted in the absence of any commercial or financial relationships that could be construed as a potential conflict of interest.
